# Suppression of Ribosome Biogenesis by Targeting WD Repeat Domain 12 (WDR12) Inhibits Glioma Stem-Like Cell Growth

**DOI:** 10.3389/fonc.2021.751792

**Published:** 2021-11-12

**Authors:** Lanjuan Mi, Qinghui Qi, Haowen Ran, Lishu Chen, Da Li, Dake Xiao, Jiaqi Wu, Yan Cai, Songyang Zhang, Yuanyuan Li, Bohan Li, Jiong Xie, Haohao Huang, Tao Li, Tao Zhou, Ailing Li, Ji Qi, Fangye Li, Jianghong Man

**Affiliations:** ^1^ State Key Laboratory of Proteomics, National Center of Biomedical Analysis, Beijing, China; ^2^ Department of Neurosurgery, Beijing Fengtai Hospital, Beijing, China; ^3^ Department of Neurosurgery, General Hospital of Central Theater Command of Chinese People’s Liberation Army, Wuhan, China; ^4^ Department of Neurosurgery, First Medical Center of PLA General Hospital, Beijing, China

**Keywords:** glioblastoma (GBM), glioma stem-like cells (GSCs), WDR12, ribosome, therapy of glioblastoma

## Abstract

Glioma stem-like cells (GSCs) are a subset of tumor cells that initiate malignant growth and promote the therapeutic resistance of glioblastoma, the most lethal primary brain tumor. Ribosome biogenesis is an essential cellular process to maintain cell growth, but its regulatory mechanism in GSCs remains largely unknown. Here, we show that WD repeat domain 12 (WDR12), a component of the Pes1-Bop1 complex (PeBoW), is required for ribosome biogenesis in GSCs. WDR12 is preferentially expressed in GSCs compared to non-stem tumor cells and normal brain cells. High levels of WDR12 are associated with glioblastoma progression and poor prognosis. Silencing WDR12 results in the degradation of PeBoW complex components and prevents the maturation of 28S rRNA, thereby inhibiting ribosome biogenesis in GSCs. Subsequently, WDR12 depletion compromises GSC proliferation, inhibits GSC-derived orthotopic tumor growth, and extends animal survival. Together, our results suggest that WDR12 is crucial for ribosome biogenesis in GSCs, and is thus a potential target for GSC-directed therapy of glioblastoma.

## Introduction

Glioblastoma multiforme (GBM) ranks among the most aggressive and lethal of human brain tumors with dismal prognoses. Despite therapeutic advances in treatments, the survival of GBM patients is usually less than 16 months (1–3). Glioma stem-like cells (GSCs) represent a subpopulation of tumor cells with the capacities of self-renewal, multilineage differentiation, and potent tumor initiation. Studies show that GSCs contribute to therapeutic resistance and tumor recurrence of gliomas, suggesting that targeting GSCs may be a promising strategy to improve GBM treatment (4–7).

The ribosome is one of the oldest molecular machines. It is composed of distinct proteins and nucleic acids and is responsible for protein synthesis in every living cell. The human 80S ribosome is composed of a large 60S subunit that harbors the 28S, 5.8S, and 5S rRNAs plus 47 proteins, and a small 40S subunit that contains the 18S rRNA and 33 proteins. The large and small subunits migrate from the nucleolus to the cytoplasm where they form the 80S ribosome. During the process, the synthesis of rRNA, but not of ribosomal proteins, is the rate-limiting step of ribosome formation (8–10). Ribosome biogenesis is responsible for the translation of information contained in mRNA into proteins. It is well-established that increased ribosome biogenesis and protein synthesis are required for sustaining tumor cell growth and proliferation (8, 10). However, the molecular mechanism by which ribosomal biogenesis is regulated in GSCs remains largely unclear.

WDR12 (WD repeat domain 12), a member of the family of proteins ending with tryptophan–aspartic acid (WD) repeats, is ubiquitously expressed during embryogenesis, but only highly expressed in the thymus and testis in adult mice (11). The biological functions of WDR12 remain largely unexplored. Recently, WDR12 has been identified as a novel member of the Pes1-Bop1 complex (PeBoW), which plays essential roles in ribosome biogenesis and cell proliferation (12, 13). Studies suggest that WDR12 functions together with Pes1 and Bop1 to promote the progression of the cell cycle and maturation of the 60S large ribosomal subunit (12–14). Thus, the expression of PeBoW components needs to be regulated to accommodate ribosome synthesis and cell growth. For example, quiescent or serum-starved cells express relatively low levels of Pes1, Bop1, and WDR12 (13). Dysregulation of Pes1 and Bop1 has been implicated in the chromosomal instability, tumorigenesis, and therapeutic resistance of different types of tumor (15–19). However, the expression and roles of WDR12 in GSCs and GBM remain to be explored.

In this study, we found that WDR12 was preferentially expressed in GSCs relative to non-stem tumor cells (NSTCs) and normal brain cells. Importantly, depletion of WDR12 significantly suppressed GSC proliferation and tumor progression. Mechanistically, our results demonstrated that WDR12 formed a complex with Pes1 and Bop1 in GSCs. Loss of WDR12 resulted in the degradation of Pes1 and Bop1 proteins and compromised the processing of 28S rRNA, thus suggesting that WDR12 is required for the maintenance of the PeBoW complex and for ribosome biogenesis in GSCs. Taken together, our study suggests that GSCs may maintain ribosome biogenesis through upregulating WDR12; thus, targeting ribosome biogenesis *via* WDR12 represents a therapeutic index for GBM treatment.

## Materials and Methods

### Cells, Tissues and Cell Culture

The GSCs (3691, 3832, 387, 4121, 456 and H2S) and NPCs (hNP1 and 16157) were gifts from University of Pittsburgh and Cleveland Clinic. All patient-derived GSCs were isolated from GBM specimens and functionally validated as previously described (20–23). Briefly, GSCs were sorted by magnetic cell sorting using the surface marker CD133 (Miltenyibiotec) from GBM tissues. GSCs were cultured in NeurobasalTM Medium (Gibco, 12348017) containing B27 supplement (Gibco, 12587010), 10 ng/ml fibroblast growth factor (βFGF) (R&D Systems, 4114-TC), 10 ng/ml epidermal growth factor (EGF) (R&D Systems, 236-EG), 1 mM sodium pyruvate (MacGene, CC007), 2 mM L-glutamine (MacGene, CC009), 100 IU/mL penicillin and 100 µg/mL streptomycin (MacGene, CC004). Specifically, 387 GSCs were derived from a primary GBM patient (76 -year old, female); 4121 GSCs were derived from a recurrent GBM patient (53-year old, male); 456 GSCs were derived from a primary GBM patient (8-year old, female). When differentiated, GSCs were cultured in DMEM (Macgene, CM10013) with 10% fetal bovine serum (FBS) (Excell Bio, FNA500) for 7 days. hNP1 and 16157 was cultured in the stem cell medium described above. Normal human astrocytes (NHA) (from Beina Chuanglian Biotechnology Institute) and HEK293 cells (from the America Type Culture Collection) were maintained in DMEM supplemented with 5 mM glucose and 10% FBS. Cells were maintained in a humidified atmosphere containing 5% CO_2_ at 37°C. GBM specimens were collected with the approval of the PLA General Hospital. Informed consent was obtained for all subjects. The glioma tissue microarray was purchased from Shanghai Outdo Biotechnology Company (HBraG180Su02).

### Orthotopic Mouse Xenografts

All animal experiments were in accordance with the NIH guide for the care of laboratory animals and with the approval of the Institutional Animal Care and Use Committee of National Center of Biomedical Analysis. Mice used in the studies were female, 4-week-old, BALB/c nude mice purchased from Beijing Vital River Laboratory Animal Technology Co., Ltd. Animal care was monitored daily by certified veterinary staff and laboratory personnel. To establish the GBM orthotopic xenografts, 5×10^4^ GSCs were implanted into the right frontal lobes of nude mice. For the survival experiments, animals were maintained until the manifestation of neurological signs were observed. All surgical procedures were performed under anesthesia by intraperitoneal injection of a ketamine and xylazine cocktail. Tissues were removed following euthanasia and were fixed in 4% paraformaldehyde for 24 hours at 4°C, followed by 30% sucrose cryoprotection for 48 hours, before they were embedded by O.C.T. compound (Sakura, 4583).

### Western Blot

Cells were lysed in M2 Buffer (20 mM Tris HCl, pH 7.6, 0.5% NP40, 250 mM NaCl, 3 mM EDTA and 3 mM EGTA), supplemented with 1×EDTA-free protease inhibitor cocktail (MedChemExpress, HY-K0010). Protein concentration was quantified by the Bradford reagent (BIO-RAD, 500-0205). Protein samples were mixed with Laemmli loading buffer and boiled for 10 minutes. Subsequently, samples were separated by SDS polyacrylamide gelelectrophoresis (SDS-PAGE), then were transferred to polyvinylidene fluoride (PVDF) membranes (PALL, BSP0161). Blots were incubated with primary antibodies overnight at 4°C followed by HRP-conjugated species-specific secondary antibodies (Jackson Immuno Research, 1:5000) at room temperature for one hour. Finally, the signals were detected by ECL luminescence reagent (Absin, abs920). The following antibodies were used at the indicated dilutions: WDR12 (1:1000, NBP1-53111, Novus), Pes1 (1:1000, b88543, Abcam), Bop1 (1:1000, 28366-1-AP, Proteintech), SOX2 (1:1000, MAB4423, Millipore), Olig2 (1:1000, sc-48817, Santa Cruz), GFAP (1:1000, Z0344, Dako), GAPDH (1:1000, 3683s, Cell Signaling).

### Immunofluorescent Staining

Cells and tissue samples were fixed with 4% formaldehyde for 20 min at room temperature followed by washing with PBS, and then permeabilized with 0.3% Triton X-100 for 5 minutes at room temperature. After blocking with blocking buffer (1xPBS including 5% BSA) for 1 hour at room temperature, samples were incubated with primary antibody in PBS with 5% BSA at 4°C overnight. The following antibodies were used at the indicated dilutions: WDR12 (1:100, ab111955, Abcam), SOX2 (1:50, sc-17320, Santa Cruz), Ki67 (1:200, 9449S, Cell Signaling). After washing three times with PBS, samples were incubated with secondary antibody for 1h at room temperature followed by incubating with hoechst (Invitrogen). Finally, slides were observed using ZeissLSM880 system.

### DNA Constructs and Production of Retrovirus

The shRNAs targeting *WDR12, Pes1*, and *Bop1* genes (synthesized from ThermoFisher) were inserted into pLKO.1 TRC vectors and pLKO-Tet-On vector. Following targeting sequences were used: sh*WDR12* #1 (5’-TGGATCTTGACTGGTTCTTAT-3’), sh*WDR12*#2 (5’-GGCAGTCTTAAGTCAACTTTG-3’); sh*Pes1*#1 (CACATCATCAAGGAACGGTAT), sh*Pes1*#2 (AGTCACTTCTCCTCCTCCTTT); sh*Bop1*#1 (ACAGCGAGGAGAGTGTGTTCT), sh*Bop1*#2 (GATAGCAAGCTGGTGTGGTTT). HEK293 cells were used to generate lentiviral particles through co-transfection of the packaging vectors pSPAX2 and pVSVG (Addgene) using a standard calcium phosphate transfection method. After 60-72 hours of medium renewal, the medium was collected and filtered through a 0.45-μm filter. The appropriate volume of 5×PEG buffer was added to the filtrate and incubated at 4°C overnight. After centrifugation at 7000g for 15 minutes, the pellets were re-suspended in 4°C PBS. The solution was then centrifuged at 12000g at 4°C for 15 minutes. Supernatant was divided into equal volumes and was used to infect GSCs.

### Cell Proliferation and Sphere Formation

Celtiter-Glo Luminescent Cell Viability Assay (Promega, G7572) was used for cell viability measurements. Forty-eight hours after lentiviral infection, the GSCs were incubated in a culture medium containing puromycin (1 µg/mL) for another 24 hours. The GSCs were isolated to the single-cell and were seeded in 96-well plates at a density of 2×10^3^ cells per well. Cell viability were examined on the 0, 2^nd^, 4^th^, and 6^th^ day, and the numbers of sphere were counted at 5^th^ day for analysis.

### Cell Cycle Analysis

The GSCs were isolated to the single-cell and were rinsed with 4°C PBS twice. After centrifugation at 1200rmp for 5 minutes, the pellets were re-suspended in 250 µl PBS. 750 µl ethanol absolute was added to the resuspension and incubated at 4°C overnight. After centrifugation at 3000rmp for 3 minutes, the pellets were rinsed with buffer (0.5% BSA and 1x PBS) twice. The pellets were re-suspended in 0.1 mg/ml PI (0.1 mg/ml RNAase and 1x PBS) and incubated at 37°C for 30 minutes in dark. Finally, cell cycle was measured by flow cytometry (FCM).

### RNA Sequencing

Total RNA was isolated from cells using Trizol (Sigma-Aldrich) and broken into small fragments. RNA-seq analysis of GSCs was performed using an Illumina HiSeq 2500 instrument at the Oebiotech Corp. Clean data were obtained by Trimmomatic. FPKMs (Fragments Per kb Per Million mapped Reads) of known genes were calculated using eXpress and Bowtie2. Differentially expressed genes were identified using the DESeq R package functions estimate Size Factors and nbinomTest.

### RNA Isolation and Real-Time PCR

Cell pellets were collected and the total RNA was extracted using RNeasy kit (QIAGEN, 74104), then reversely transcribed to cDNA with PrimeScriptTM RT Master Mix (Takara Bio Inc. RR036A) according to the manufacturer’s instructions. Real-time PCR was performed with SYBR Green Master Mix (Applied BiosystemsTM, A25778) on a cycler (Applied BiosystemsTM). GAPDH or Actin was used for normalization. The primer pairs used to detect the mRNA levels are listed in **Table 1**.

**Table 1:** Primer sequences used for Real-Time PCR.

SOX2	Forward 5’-GCCGAGTGGAAACTTTTGTCG-3’
	Reverse 5’-GGCAGCGTGTACTTATCCTTCT-3’
Olig2	Forward 5’-CAAGAAGCAAATGACAGAGCCGGA-3’
	Reverse 5’-TGGTGAGCATGAGGATGTAGTTGC-3’
GFAP	Forward 5’-GAGCCTCAAGGACGAGATGG-3’
	Reverse 5’-CCAGGCTGGTTTCTCGAATCT-3’
GAPDH	Forward 5’-CCAGGTGGTCTCCTCTGACTTC-3’
	Reverse 5’-GTGGTCGTTGAGGGCAATG-3’
WDR12	Forward 5’- AGCTCCAAACACGCTTCTACA -3’
	Reverse 5’- AGGGCATTCGCAGAAACTGG -3’
Pes1	Forward 5’- GGCCACCAACTACATCACCC -3’
	Reverse 5’- AGAATGCACAGCCGCCTAAA -3’
Bop1	Forward 5’- ATGGCAGGCGCATCTACAAG -3’
	Reverse 5’- CTCATCCGTCAGTCTCAGGTC -3’

### Northern-Blot

A population of RNA was isolated from cell sample using Trizol. The different size of RNA was separated by denaturing agarose gel electrophoresis. The RNA was transferred to a positively charged nylon membrane and then immobilized for subsequent hybridization. After prehybridization and hybridization, unhybridized probe was removed by washing in several changes of buffer. Finally, the signals were detected.

### Spectrometry Analysis

For the WDR12 binding proteins identification, GSCs were transduced with Flag- WDR12 or vector control through lentiviral infection. Cells were collected and lysed in NP-40 lysis buffer (Boster biological technology, AR0107) supplemented with protease inhibitors, incubated on ice for 30 minutes, and followed by centrifugation at 15,000g for 15 minutes at 4°C. The supernatant was subjected to immunoprecipitation with the anti-Flag M2 beads (10 μl, A2220, Sigma-Aldrich) overnight at 4°C. The WDR12 complexes were pulled down by anti-Flag-M2 beads and separated on SDS-PAGE followed by silver staining. Gel fragments were excised, detained in 50% ethanol and 5% acetic acid, dehydrated in acetonitrile, dried in a Speed vacuum, and digested with trypsin. The peptides were extracted from the polyacrylamide and subjected to LC-MS analysis.

### Immunoprecipitation Assay

Cells were collected and lysed in NP-40 lysis buffer (Boster biological technology, AR0107) supplemented with protease inhibitors, incubated on ice for 30 minutes, and followed by centrifugation at 15,000g for 15 minutes at 4°C. The supernatant was subjected to immunoprecipitation with the anti-Flag M2 beads (10 μl, A2220, Sigma-Aldrich) overnight at 4°C. The precipitants were extensively washed with lysis buffer, boiled with SDS loading buffer and subjected to SDS-PAGE.

### Immunohistochemistry

The human glioma tissue microarrays (TMA) slides were subjected to dewaxing, rehydration, and heat-induced antigen retrieval. After blocking with 10% goat serum (ZSGB-BIO, ZLI-9022), slides were incubated with WDR12 (Abcam, ab252878, 1:100) overnight at 4°C. After incubation with secondary antibody, slides were incubated with hematoxylin (ZSGB-BIO, ZLI-9610) to stain the cell nuclei. Staining was visualized using 3, 30-diaminobenzidine (DAB) chromogen (Zhongshan Golden Bridge). Finally, images were taken under a microscope. Histologic diagnosis of the tissue microarray was reviewed by at least 2 individuals, one of whom is a pathologist. And consensus scores are reported. The staining intensity of WDR12 has assessed both the intensity of the staining and the percentage of positively stained cells. For the intensity, a score of 0–3 (corresponding to negative, weak, moderate, or strong staining) was recorded and the percentage of positively stained cells at each intensity was estimated.

### Statistical Analysis

All the results in the figures are expressed as means ± standard deviation (SD), and all analyses used GraphPad Prism 8.1. Results were considered statistically significant at p < 0.05, and the p values are as follows: *p < 0.05, **p < 0.01, and ***p < 0.001. Gene Set Enrichment Analysis (GSEA) was used to examine differentially expressed genes (DEGs). The output of GSEA is a normalized enrichment score (NES) which accounts for the size of the gene set being tested. The Cytoscape plugins EnrichmentMap was used to generate gene set enrichment bubble plots with FDR q value threshold of 0.05 as default. The unpaired Student’s t-test (two-tailed) or Welch’s t test was used for the comparison between unpaired two-groups. For the survival analysis, Kaplan-Meier survival curves were analyzed by using log-rank statistics comparing the different patient or mouse groups.

## Results

### WDR12 Is Preferentially Upregulated in Glioma Stem-Like Cells

**Figure 1:**
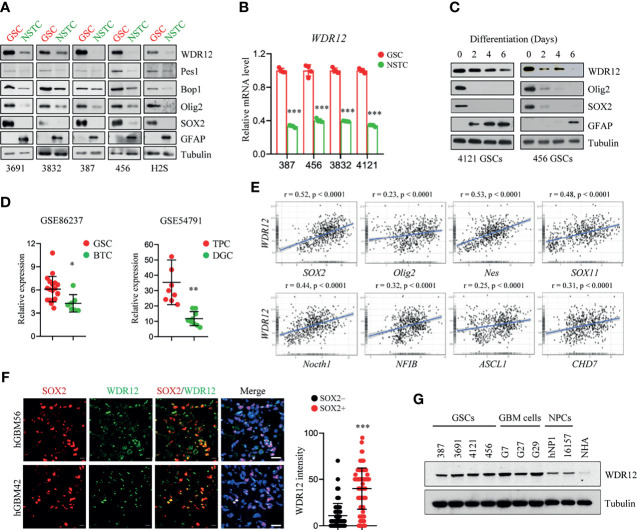
WDR12 is preferentially upregulated in Glioma Stem-like Cells. **(A)** Immunoblot (IB) showing the expression of WDR12, Pes1, Bop1, SOX2, Olig2, GFAP, and GAPDH in GSCs and matched NSTCs. **(B)** Relative mRNA expression of *WDR12* was analyzed by qRT-PCR in GSCs and matched NSTCs. **(C)** IB analysis the expression of WDR12, SOX2, Olig2, and GFAP during serum-induced GSC differentiation. **(D)** Relative mRNA expression of *WDR12* in GSCs (n=19) relative to bulk tumor cells (BTCs, n=7) from GEO profile (GSE86237, left), and in tumor propagating cells (TPCs, n=9) relative to differentiated glioma cells (DGCs, n=9) from GEO profile (GSE54791, right) are shown. **(E)** Pairwise correlation analysis of the indicated genes in REMBRANT database. Pearson correlation coefficient (r) value and P value are shown. **(F)** Representative immunoflourescent images of human primary GBM specimens stained with anti-WDR12 (green) and anti-SOX2 (red). Nuclei were counterstained with Hoechst (blue) (left). Scale bars, 20 μm. Quantifications of WDR12 staining intensity in SOX2+ (n=113) and SOX2- (n=130) cells (6 random microscope fields from 4 tumors) are shown (right). **(G)** IB showing the expression of WDR12 and Tubulin in GSCs, isolated primary GBM cells, human neural progenitor cells (hNP1 and 16157) and normal human astrocyte (NHA). Data are represented as mean ± SD. *p < 0.05, **p < 0.01, ***p < 0.001, as assayed by Unpaired Student’s t-test or Welch’s t test.

In our previous mass spectrometry screen, we identified a series of proteins, including WDR12, that were highly expressed in glioma stem-like cells (GSCs) compared to those in non-stem tumor cells (NSTCs) (24). To validate the upregulation of WDR12 in GSCs, we first performed an immunoblot (IB) analysis in multiple GSC lines, which have been functionally validated in previous studies (20, 25, 26), and their matched NSTCs. We observed that protein levels of WDR12 were dramatically increased in all five GSC lines compared to that in NSTCs (**Figure 1A**). However, the protein levels of Pes1 and Bop1, two components of the PeBoW complex, were slightly increased in GSCs relative to those in NSTCs (**Figure 1A**). Putative stem cell markers, such as the sex determining region y-box 2 (SOX2) and oligodendrocyte lineage transcription factor 2 (Olig2), were increased in GSCs. The differentiated marker, glial fibrillary acidic protein (GFAP), was increased in NSTCs (**Figure 1A**). Transcriptional expression of the *WDR12* gene was validated using quantitative real-time polymerization chain reaction (qRT-PCR) in four GSCs and their matched NSTCs (**Figure 1B** and **Supplementary Figure 1A**). In addition, we found that the levels of WDR12 were gradually decreased during GSC differentiation induced by serum treatment (**Figure 1C**). Meanwhile, we observed the reduction of SOX2 and Olig2, and the induction of GFAP upon serum treatment, thus confirming the differentiated status of GSCs (**Figure 1C**).

To further determine the expression of WDR12 in GSCs, we queried the Gene Expression Omnibus (GEO) databases GSE86237, which includes 18 GSCs and 7 bulk tumor cells (BTCs) isolated from human GBM patients (27), and GSE54791, which includes 3 stem-like tumor-propagating cells (TPCs) and their matched differentiated glioma cells (DGCs) (28). Consistent with our above results, WDR12 was significantly upregulated in GSCs and TPCs relative to that in BTCs and DGCs respectively (**Figure 1D**). Moreover, we analyzed the Repository of Molecular Brain Neoplasia Data (REMBRANDT) (29) and the Chinese Glioma Genome Atlas (CGGA) (30) (http://www.cgga.org.cn) databases, and we found that WDR12 was significantly and positively correlated with genes encoding stem cell markers, including *Sox2*, *Olig2*, *Nes*, *Sox11*, *Notch1*, *NFIB*, *ASCL1* and *CHD7*, in both glioma databases (**Figure 1E** and **Supplementary Figure 1B**).

We next assessed the localization of WDR12 in GSCs by performing co-immunofluorescent (co-IF) staining. WDR12 was found to be localized mainly in the nucleolus in GSCs (**Supplementary Figure 1C**), which is consistent with previous reports that WDR12 is involved in the assembly of the PeBoW complex in the nucleolus (12, 13). To investigate the expression of WDR12 in GSCs *in vivo*, we performed the co-IF staining with WDR12 and SOX2 in human primary GBM specimens. The results showed that expression of WDR12 was strongly increased in tumor cells expressing SOX2 (**Figure 1F**). Additionally, we found that WDR12 was upregulated in the primary tumor cells isolated from GBM patients, as well as in GSCs, compared to that in the human neural progenitor cells including hNP1 and 16157, and normal human astrocyte (NHA) (**Figure 1G**). Taken together, these data suggest that WDR12 is preferentially upregulated in GSCs.

### Depletion of WDR12 Inhibits GSC Cell Proliferation

**Figure 2:**
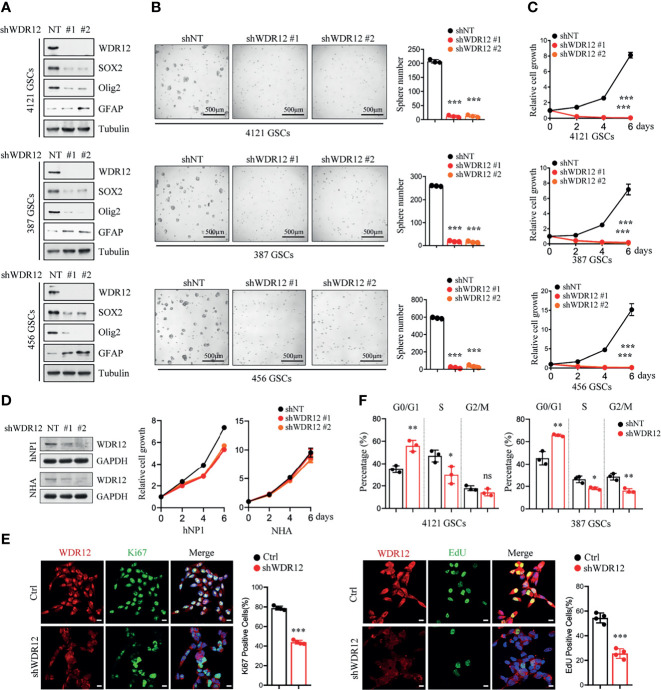
Depletion of WDR12 inhibits GSC cell proliferation. **(A)** GSCs (4121, 387, and 456) were transduced with two different shWDR12 sequences for 24 hours. IB showing the levels of indicated proteins in Ctrl and WDR12 knocked down GSCs. **(B)** Quantifications of tumor sphere numbers (2,000 cells/well) formed by Ctrl or WDR12 knocked down for 5 days in GSCs (right). Representative images of tumor spheres are shown (left). **(C)** GSCs (4121, 387 and 456) transduced with shWDR12 resulted in a decrease in cell viability, as measured by cell titer assay. **(D)** Knockdown of WDR12 had limited effects on cell growth of hNP1 and NHA, as measured by cell viability assay. IB showing the efficiency of WDR12 knockdown (left). **(E)** Knockdown of WDR12 inhibited GSC proliferation. Ki67 staining (left) and EdU incorporation assay (right) were performed in 4121 GSCs transduced with shNT or shWDR12 for 24 hours. Representative images are shown (left). The percentages of Ki67+ and EdU+ cells were quantified (right, 4 random microscope fields). Scale bars, 10 μm. **(F)** Cell cycle analysis were performed by flow cytometry in 4121 and 387 GSCs transduced with shNT or shWDR12 for 24 hours. Data are represented as mean ± SD. *p < 0.05, **p < 0.01, ***p < 0.001, as assayed by Unpaired Student’s t-test or Welch’s t test. ns, no sense.

To determine the functions of WDR12 in GSCs, we knocked down the expression of WDR12 using two independent small hairpin RNA (shRNA) sequences in three GSC lines. The efficiency of WDR12 knockdown was evaluated by IB and Q-PCR (**Figure 2A** and **Supplementary Figure 2A**). The results showed that knockdown of WDR12 resulted in a decrease in SOX2 and Olig2 expression and an increase in GFAP expression in different GSC lines (**Figure 2A**). Moreover, depletion of WDR12 strongly inhibited GSC tumor-sphere formation (**Figure 2B**), thus indicating that WDR12 might be required for GSC maintenance. Additionally, we found that silencing WDR12 significantly decreased cell viability of GSCs (**Figure 2C**). However, knockdown of WDR12 resulted in little impact on NHA and hNPC (human neural progenitor cell) viability (**Figure 2D**).

To further evaluate the biological effects of WDR12 on GSCs, we investigated cell proliferation and cell apoptosis in GSCs with or without WDR12 knocked down. Because knockdown of WDR12 for long period caused significant suppression in cell viability (**Figure 2C**), the shRNAs were expressing for 24 hours to assess cell proliferation and apoptosis, and about 50% of GSCs expressing shWDR12 were viable at this time point (**Supplementary Figure 2B**). The results showed that silence of WDR12 dramatically inhibited GSC proliferation, which was assessed by Edu (5-ethynyl-29-deoxyuridine) incorporation and Ki67 staining *in vitro* (**Figure 2E** and **Supplementary Figure 2C**). However, we did not observe any significant increase in cleaved-caspase 3 in WDR12-depleted GSCs (**Supplementary Figure 2D**), thus indicating that WDR12 has no effect on GSC apoptosis. We then performed a flow cytometry analysis and found that knockdown of WDR12 resulted in a significant induction of cells in the G0/G1 phase, but a decrease in cells in S phase in GSCs (**Figure 2F**). These results suggest that blockade of WDR12 expression suppresses GSC proliferation.

### WDR12 Is Required for GSC rRNA Processing

**Figure 3:**
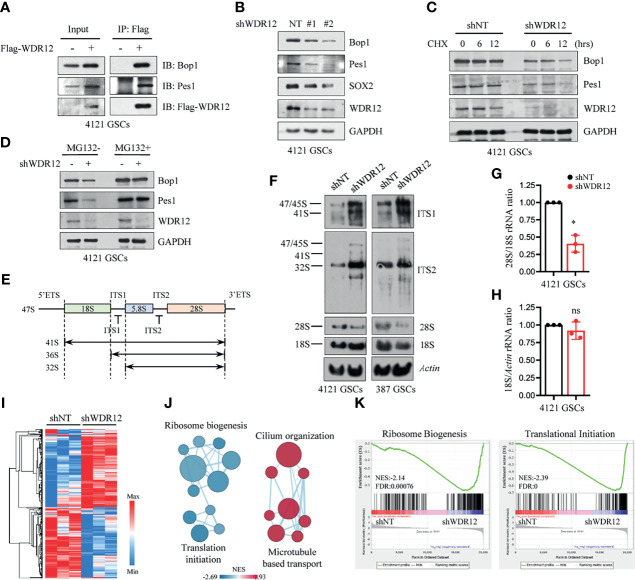
WDR12 is required for GSC rRNA processing. **(A)** Co-immunoprecipitation (Co-IP) with anti-Flag beads in Flag-vector and Flag-WDR12 expressing GSCs and IB for WDR12, Pes1 and Bop1 are shown. **(B)** IB showing that knockdown of WDR12 decreased the protein levels of Pes1 and Bop1 in 4121 GSCs. **(C)** IB showing the CHX (50 μg/ml) chase analysis of Pes1 and Bop1 protein degradation at indicated time points in GSCs with or without WDR12 knockdown. **(D)** IB showing the levels of Pes1, Bop1 and WDR12 in shNT or shWDR12 transduced GSCs treated with vehicle control or MG132 (10 μM) for 12 hours. **(E)** Diagram of the primary 47S rRNA transcript and the major rRNA intermediates. Positions of the hybridization probes are depicted. ETS, external transcribed spacer; ITS, internal transcribed spacer. **(F)** Knockdown of WDR12 impairs formation of mature 28S rRNA. Northern blot analysis of total RNA extracted from GSCs after WDR12 knockdown. Biotin labeled probes binding to the ITS1, ITS2, 18S or 28S were used to detect the indicated rRNA precursors. Actin as loading control. **(G, H)** Ratio of 28S/18S rRNA **(G)** and 18S/Actin **(H)** in 4121 GSCs transduced with shNT or shWDR12. Quantification of three independent experiments as shown. Data are represented as mean ± SD. *p < 0.05, as assayed by Welch’s t test. **(I–K)** RNA-seq analysis in 4121 GSCs transduced with shNT or shWDR12 for 24 hours. Heatmap showing relative expression levels of genes down- or up-regulated in shWDR12-GSCs compared with the shNT-GSCs. A relative color scheme used the minimum and maximum values in each row to convert values to colors **(I)**. Pathway enrichment analysis using GSEA **(K)**, and visualization using Cytoscape Enrichment Map **(J)** in GSCs with WDR12 knocked down are shown. Red nodes represent upregulated pathways and blue nodes represent downregulated pathways in WDR12 knocked down GSCs compared to control GSCs (FDR < 0.05). ns, no sense.

To explore the molecular mechanism by which WDR12 promotes GSC proliferation, we performed immunoprecipitation with Flag-tagged WDR12 and subsequent mass spectrometry to identify putative binding proteins of WDR12 in GSCs. The results showed that Pes1 and Bop1, the other two components of the PeBoW complex, were the top two binding partners of WDR12 in GSCs (**Supplementary Figure 3A**). Previous studies suggest that WDR12 is a member of the PeBoW complex and is implicated in ribosome biogenesis and cell proliferation (13). Because GSCs possess high levels of WDR12, we speculate that WDR12 may play key roles in ribosome biogenesis in GSCs. We first assessed the association between WDR12 and Pes1/Bop1 by performing a co-immunoprecipitation assay, and we found that WDR12 strongly interacted with Bop1 and Pes1 in GSCs (**Figure 3A**). Interestingly, we noticed that the expression of Bop1 and Pes1 were induced by Flag-WDR12 overexpression in the cell lysates of GSCs (**Figure 3A**). To investigate whether the levels of Bop1 and Pes1 were affected by WDR12, we knocked down WDR12 expression in GSCs. The results showed that depletion of WDR12 decreased the protein levels, but not the mRNA expression of Bop1 and Pes1 in GSCs (**Figure 3B** and **Supplementary Figure 3B**), thus indicating that WDR12 may be required for the stability of the PeBoW complex in GSCs.

To determine whether WDR12 accounts for the protein stability of the PeBoW complex, we performed a cycloheximide (CHX) chase assay to examine the half-life of Bop1 and Pes1 in GSCs with and without WDR12 depletion. Bop1 and Pes1 proteins were stable in GSCs, for the reason that CHX treatment for 12 hours had no obvious effect on the stability of these two proteins. However, WDR12 depletion resulted in a significant decrease in the half-life of Bop1 and Pes1 proteins in GSCs, but not in that of GAPDH (**Figure 3C**). To assess the potential mechanism associated with the degradation of Bop1 and Pes1 in GSCs, we treated GSCs with either MG132, an inhibitor of proteasome, or chloroquine, an inhibitor of lysosome acidification. MG132 treatment increased Bop1 and Pes1 protein levels in a time-dependent manner, but chloroquine treatment caused no appreciable change in either protein (**Supplementary Figure 3C**). Notably, MG132 treatment largely rescued the decrease of Bop1 and Pes1 in WDR12 depleted GSCs (**Figure 3D**), suggesting that loss of WDR12 might result in proteasomal degradation of Bop1 and Pes1.

To investigate the role of WDR12 in rRNA processing, we used northern blot analysis to assess the levels of different rRNA species in GSCs with or without WDR12 depletion. Total RNA was probed with sequences specific to ITS1 and ITS2 (internal transcribed spacer) of the rRNA intermediates (**Figure 3E**). The results showed that knockdown of WDR12 resulted in a remarkable accumulation of the 32S rRNA precursor (**Figure 3F**). However, the production of mature 28S rRNA was dramatically reduced, resulting from inefficient processing of the 32S rRNA precursor (**Figure 3F**), as ascertained from the decreased ratio of 28S/18S rRNA (**Figure 3G**). In addition, knockdown of WDR12 did not affect the synthesis of mature 18S rRNA, which was determined by the signal intensity of 18S rRNA normalized to Actin (**Figure 3H**). However, depletion of WDR12 only resulted in a slightly decrease in the production of 28S rRNA in hNP1 and NHA (**Supplementary Figure 3D**).

To better understand the role of WDR12, we performed RNA sequencing (RNA-seq) analysis of GSCs with and without WDR12 depletion. Knockdown of WDR12 resulted in robust changes in gene expression (**Figure 3I**). Gene set enrichment analysis (GSEA) coupled to EnrichmentMap visualization (31) showed that knockdown of WDR12 primarily resulted in a significant enrichment of signaling pathways linked to downregulation of ribosome biogenesis and translation initiation, and upregulation of cilium organization and microtubule-based transport (**Figures 3J, K**), thus supporting the major roles of WDR12 in ribosome biogenesis in GSCs. To further assess the biological function of PeBoW complex in GSCs, we knocked down Bop1 expression using two independent shRNA sequences. The results showed that silencing Bop1 consistently decreased 28S rRNA production in GSCs (**Supplementary Figure 3E**). In addition, downregulation of Pes1 or Bop1 inhibited cell viability in GSCs (**Supplementary Figure 3F**), thus supporting the important role of PeBoW complex in GSC ribosome biogenesis and growth. Together, our results showed that WDR12 was preferentially upregulated in GSCs among these three components of PeBoW complex, suggesting that WDR12 may be a specific therapeutic target to block the ribosome biogenesis in GSCs.

### WDR12 Promotes GSC-Derived Tumor Growth

**Figure 4:**
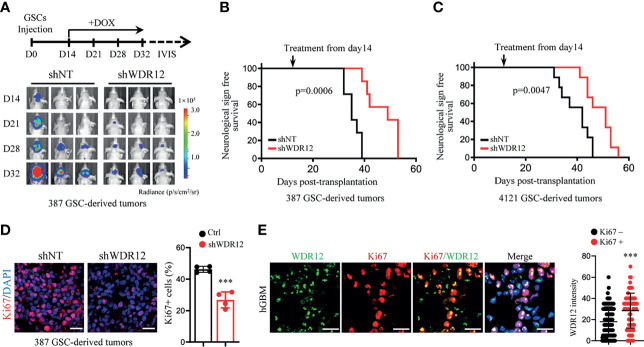
WDR12 promotes GSC-derived tumor growth. **(A–D)** Luciferase-labeled GSCs were transduced with the inducible-shWDR12 or -shNT and then transplanted into the brains of nude mice (2 × 10^4^ cells/mouse). Mice were randomly grouped and treated with Dox (1mg/ml in drinking water) at day 14 after implantation, as showed by schematic representation (**A**, top). 387 GSC-derived GBM tumors were tracked by bioluminescence and the representative images are shown (bottom) **(A)**. Kaplan-Meier survival plots of mice are shown. 387 GSCs groups, n=7 for each group; 4121 GSCs, n=9 for each group **(B, C)**; Log rank test. IF staining of Ki67 in GBM tumors derived from 387 GSCs expressing inducible-shNT or inducible-shWDR12 (left). Scale bars, 20μm. The percentage of Ki67+ cells was quantified (right, n = 4) **(D)**. **(E)** Co-IF staining of WDR12 (green) and Ki67 (red) in human GBM specimens are shown. Nuclei were counterstained with Hoechst (blue) (left). Scale bars, 20μm. Quantifications of WDR12 staining intensity in Ki67+ (n=106) and Ki67- (n=249) cells (4 random microscope fields from 4 tumors) are shown (right). Data are represented as mean ± SD. ***p < 0.001, as assayed by Unpaired Student’s t-test.

Studies suggest that GSCs have an enhanced capacity for fueling tumor growth and therapeutic resistance (22, 32, 33). To explore the effect of WDR12 *in vivo*, we employed a doxycycline (Dox)-inducible shRNA system to silence WDR12 expression and evaluated its role in GSC-derived tumor growth. A pool of GSCs stably expressing the luciferase reporter and Dox-inducible short hairpin non-targeting control RNA (shNT) or shWDR12 were generated. Expression of WDR12 was significantly decreased in Dox-shWDR12 GSCs upon Dox treatment (**Supplementary Figure 4A**). Subsequently, Dox treatment potently inhibited tumorsphere formation and reduced cell viability in Dox-shWDR12 GSCs (**Supplementary Figures 4B, C**). Next, we intracranially implanted GSCs expressing the luciferase reporter plus Dox-shNT or Dox-shWDR12 into immunocomprised mice and treated them with Dox from day 14 after implantation (**Figure 4A**, up). The results showed that inducible knockdown of WDR12 inhibited GSC derived tumor growth, as shown by bioluminescent imaging (**Figure 4A**, bottom), and significantly prolonged animal survival (**Figures 4B, C**). The knockdown efficiency was evaluated by IF staining of WDR12 in the GSC-derived tumor tissues harvested from animals at day 28 (**Supplementary Figure 4D**). In addition, we observed that silencing WDR12 in tumors remarkably decreased tumor cell proliferation and GSC population, as assessed by Ki67 and SOX2 staining respectively (**Figure 4D** and **Supplementary Figure 4D**). In human GBM specimens, expression of WDR12 was increased in Ki67+ tumor cells (**Figure 4E**), thus suggesting a correlation of WDR12 levels with tumor cell proliferation. Together, these data suggest that WDR12 is required for GSC proliferation and GBM tumor growth.

Notably, although depletion of WDR12 *in vivo* significantly extended the survival, mice eventually succumbed to tumors. We performed IF staining with GSC-derived tumor tissues and observed the re-expression of WDR12 in a fraction of tumor cells in the dying mice treated with Dox at day 40 (**Supplementary Figure 4D**). These results indicate that the efficiency of inducible-knockdown of WDR12 may be compromised by long-term *in vivo* administration of Dox. Consistent with the re-upregulation of WDR12, the fraction of SOX2 positive cells was also increased in tumors of the dying mice (**Supplementary Figure 4D**).

**Figure 5:**
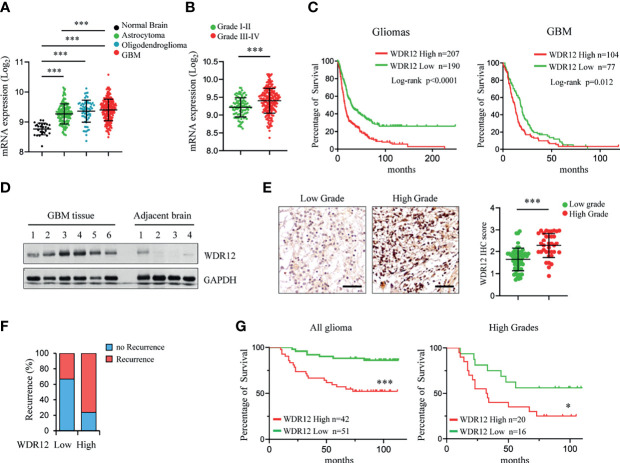
Expression level of WDR12 is associated with poor survival in glioma and GBM. **(A)** Plots of WDR12 expression in normal brains, astrocytoma, oligodendroglioma and GBM from REMBRANDT glioma database. **(B)** Plots of WDR12 expression in grades I-II and grades III-IV gliomas from REMBRANDT glioma database. **(C)** Kaplan-Meier survival analysis of glioma and GBM patients from REMBRANDT glioma database stratified by WDR12 expression. **(D)** IB showing the levels of WDR12 in human primary GBM tissues and adjacent normal brain tissues. **(E–G)** IHC analysis of WDR12 in a glioma tissue microarray. Representative images and plots of histoscore of WDR12 in low grades and high grades gliomas are shown **(E)**. Scale bars, 50 μm. (Low grades, n=57; High grades, n=36). The percentages of recurrence of gliomas in tumors with low (n=51) or high (n=42) expression of WDR12 are shown **(F)**. Kaplan-Meier survival analysis of patients with WDR12 low and WDR12 high expression in all gliomas (left) and high grade gliomas (right) are shown **(G)**. (Log rank test). Data are represented as mean ± SD. ***p < 0.001, as assayed by Unpaired Student’s t-test or Welch’s t test. *p < 0.05, ***p < 0.001.

To evaluate the clinical relevance of WDR12 in glioma and GBM, we analyzed the REMBRANDT and Gravendeel brain neoplasia databases (29, 34). The expression of WDR12 and Pes1 were both significantly upregulated in astrocytoma, oligodendroglioma, and GBMs compared to that in normal brains (**Figure 5A** and **Supplementary Figures 5A, D**). The expression of Bop1 was not available in the REMBRANDT database. However, the levels of WDR12, but not of Pes1, were higher in high-grade gliomas than that in low-grade gliomas (**Figure 5B** and **Supplementary Figures 5B, E**). Moreover, the expression of WDR12 was significantly inversely correlated with survival of both gliomas and GBMs (**Figure 5C** and **Supplementary Figure 5C**). However, high levels of Pes1 were not significantly correlated with poor outcome in GBM (**Supplementary Figure 5F**). To better address the clinical relevance of WDR12, we performed an IB analysis with fresh GBM specimens and confirmed the elevated expression of WDR12 in tumor tissues relative to normal brain tissues adjacent to tumors (**Figure 5D**). Immunohistochemically (IHC) staining with WDR12 antibody in a human glioma tissue microarray showed that both the intensity of WDR12 staining and the percentage of WDR12 positive cells were strongly increased in high-grade gliomas compared to low-grade gliomas (**Figure 5E**). Additionally, glioma patients with high levels of WDR12 showed increased recurrence (**Figure 5F**). Importantly, high expression of WDR12 was associated with poor survival in all gliomas, whether high- or low-grade (**Figure 5G**). Together, these results suggest that WDR12 is highly expressed in GBMs and associated with the malignant progression of gliomas.

## Discussion

Glioma stem-like cells (GSCs) have been implicated in the treatment failure of glioblastoma (GBM), an incurable primary brain tumor, because of their self-renewal properties, high tumorigenic potential, and resistance to standard cytotoxic therapy (5, 7, 32, 33). Due these capacities, GSCs represent critical drug targets. However, a GSC-targeting drug is still unavailable for clinical practice. An understanding of the mechanism by which GSCs co-opt core developmental programs to maintain their growth and survival is urgently needed.

Ribosome biogenesis is a fundamental process that is responsible for the translation of information contained in mRNA into functional proteins. There is growing evidence indicating that accelerated ribosome biogenesis is associated with malignant transformation (8, 35). Human 80S ribosome is composed of a 60S and a 40S subunit. Studies demonstrate that Pes1, Bop1, and WDR12 form the PeBoW complex in mammalian cells and promote pre-rRNA processing for the synthesis of 28S and 5.8S rRNAs to assemble the 60S large ribosomal subunit (13). Biological functions of components of the PeBoW complex, such as Pes1 and Bop1, have been implicated in multiple types of malignant tumor progression, including breast cancer, colon cancer, liver cancer, and melanoma (15–17, 36). A recent study has shown that WDR12 may be an oncogene in GBM (37), however, the molecular mechanisms underlying the promotion of WDR12 in GBM progression remain to be further explored. In our study, we found that WDR12 is significantly upregulated in glioma and GBM, and is associated with poor outcomes of brain tumors, indicating that WDR12 may be a prognostic marker in glioma and GBM.

Notably, our finding demonstrate that WDR12 is preferentially expressed in GSCs compared with NSTCs. Glioma databases analysis shows a strong positive correlation between the expression of WDR12 and multiple stem cell markers, suggesting that tumors with high levels of WDR12 may be enriched in GSCs. Importantly, depletion of WDR12 decreases the processing of 32S rRNA and the subsequent maturation of 28S rRNA in GSCs, thereby inhibiting GSC proliferation and tumor growth. Additionally, RNA-seq analysis demonstrates that depletion of WDR12 results in a dramatic enrichment in the downregulation of pathways linked to ribosome biogenesis and protein translation. Interestingly, we observed that silencing WDR12 shows no significant impact on rRNA processing and cell growth in normal brain cells, which may due to the low level of WDR12 in these cells. Moreover, normal brain cells grow much slower compared with tumor cells, that may result in the insensitive of normal brain cells to ribosomal suppression. These findings reveal that high expression of WDR12 is specific required for the ribosome biogenesis in GSCs. Additionally, our studies show that reducing WDR12 expression results in the proteasome-depended degradation of Pes1 and Bop1 in GSCs. These results suggest that WDR12 is required for the stability and maintenance of the PeBoW complex in GSCs.

While we present evidence that downregulating WDR12 suppresses tumor growth in GBM mouse model, a fraction of GSCs are still remained in the tumors. Additional studies using CRISPR-Cas9 system to completely knockout WDR12 *in vivo* will be crucial to establishing the role of WDR12 in GSC maintenance *in vivo* and in GSC-derived tumor propagation. Moreover, it remains to be further explored whether WDR12 is also required for the stabilization of the PeBoW complex in other cell types and how WDR12 protects the components of this composite from proteasomal degradation.

Collectively, we found that WDR12 is highly expressed in GSCs and is essential for ribosome biogenesis and cell proliferation of GSCs, suggesting that GSCs may maintain high ribosome activity through upregulating WDR12. Targeting WDR12 may be a promising therapeutic opportunity to compromise ribosome function and inhibit GSC derived tumor progression.

## Data Availability Statement

The datasets presented in this study can be found in online repositories. The names of the repository/repositories and accession number(s) can be found below: https://ngdc.cncb.ac.cn/gsa-human/browse/HRA001129.

## Ethics Statement

All animal experiments were performed in accordance with the NIH guide for the care and use of laboratory animals and with the approval of the Institutional Animal Care and Use Committee of National Center of Biomedical Analysis.

## Author Contributions

LM and JM developed the working hypothesis, scientific concept and designed the experiments. JM, FL, and JQ supervised the project, analyzed data and prepared the manuscript. LM, QQ, HR, DL, DX, JW, YC, SZ, YL, LC, and HH. performed the experiments. JM, JW, and LM. performed database analyses. BL, JX, JQ, and FL provided GBM samples. AL, TZ, and TL provided scientific advice for the manuscript. All authors contributed to the article and approved the submitted version.

## Funding

This research was supported by grants from the National Key R&D Program of China (2017YFA0505602), National Natural Science Foundation of China (no. 81872408).

## Conflict of Interest

The authors declare that the research was conducted in the absence of any commercial or financial relationships that could be construed as a potential conflict of interest.

## Publisher’s Note

All claims expressed in this article are solely those of the authors and do not necessarily represent those of their affiliated organizations, or those of the publisher, the editors and the reviewers. Any product that may be evaluated in this article, or claim that may be made by its manufacturer, is not guaranteed or endorsed by the publisher.
